# Design of Strain-Engineered GeSn/GeSiSn Quantum Dots for Mid-IR Direct Bandgap Emission on Si Substrate

**DOI:** 10.1186/s11671-018-2587-1

**Published:** 2018-06-07

**Authors:** Reem Al-Saigh, Mourad Baira, Bassem Salem, Bouraoui Ilahi

**Affiliations:** 1 King Saud University Department of Physics and Astronomy, College of Sciences, Riyadh, 11451 Saudi Arabia; 2 University of Monastir Faculty of Sciences, Laboratory of Micro-Optoelectronic and Nanostructures, 5019 Monastir, Tunisia; 3 Univ. de Grenoble Alpes, CNRS, CEA/LETI Minatec, LTM, F-38000 Grenoble, France

**Keywords:** GeSn, GeSiSn, Quantum dots, Direct bandgap, Mid-IR

## Abstract

Strain-engineered self-assembled GeSn/GeSiSn quantum dots in Ge matrix have been numerically investigated aiming to study their potentiality towards direct bandgap emission in the mid-IR range. The use of GeSiSn alloy as surrounding media for GeSn quantum dots (QD) allows adjusting the strain around the QD through the variation of Si and/or Sn composition. Accordingly, the lattice mismatch between the GeSn quantum dots and the GeSiSn surrounding layer has been tuned between − 2.3 and − 4.5% through the variation of the Sn barrier composition for different dome-shaped QD sizes. The obtained results show that the emission wavelength, fulfilling the specific QD directness criteria, can be successively tuned over a broad mid-IR range from 3 up to7 μm opening new perspectives for group IV laser sources fully integrated in Si photonic systems for sensing applications.

## Background

Recently, the demonstration of direct bandgap group IV materials through the alloying of Ge [1, 2] and SiGe [3, 4] with Tin has motivated intense research activities owing to the real and practically implementable opportunities towards photonics and electronics efficient on-chip integration. Indeed, GeSn alloy has been shown to exhibit direct bandgap beyond certain composition through the faster decrease of Γ compared to L valley [5–8]. While the reported results are very encouraging, the material properties and application potentialities are not yet fully explored. Indeed, the main actually available path to increase the operating wavelength of GeSn-based semiconductor lasers, towards the atmosphere transparency window that overlaps with absorbing lines of various gases [9], includes the increase of Sn content in the GeSn layers [10, 11]. However, because of the large lattice mismatch between Ge and Sn (14%), the preservation of the crystallographic quality of the material appears as the main challenge prohibiting this goal [12, 13]. A potentially interesting solution to increase the emission wavelength and ensure better carrier confinements relay on lower dimensional structures such as nanowires [14–16], nanorods [17], and quantum dots [18]. Within the specific directness criteria, the direct bandgap interband emission wavelength is theoretically limited to 4.3 μm [19]. To overcome these limitations, it is necessary to introduce an additional degree of freedom in the conception of group IV-based quantum structures. This can be ensured by using ternary GeSiSn layer [20–22], as a surrounding material for GeSn quantum dots (QD) offering the possibility of strain engineering by incorporating appropriate Si and Sn compositions*.* Accordingly, the use of GeSiSn strain engineering layer around GeSn QD is expected to offer a larger range of accessible direct bandgap emission wavelength.

In this context, we report on theoretical study of the effect of strain engineering by varying the Sn composition in the GeSiSn layer surrounding the GeSn QD on the direct bandgap interband emission wavelength.

## Methods

Since the band offsets between binary and ternary Sn-containing group-IV alloys and Ge are not experimentally known, the relative band alignment between the different group-IV semiconductors involved in this work is evaluated, with respect to the valence band edge of Ge, using Jaros’ simplified theory of band offsets [23] as detailed by D’Costa et al. [24]. The strain effects arising from the lattice mismatch between Ge substrate and GeSiSn layer and between the GeSn QD and the surrounding GeSiSn material have been evaluated for the conduction and valence band edges.

Indeed, the conduction band edge is shifted by $\delta {E}_c^i$ and that of the valence band by *δE*_*v*_ as shown in Eq. (1) and (2):

1 $$ \delta {E}_c^i={a}_c^i\left({\varepsilon}_{xx}+{\varepsilon}_{yy}+{\varepsilon}_{zz}\right) $$

2 $$ \delta {E}_v={a}_v\left({\varepsilon}_{xx}+{\varepsilon}_{yy}+{\varepsilon}_{zz}\right)+b\left({\varepsilon}_{xx}-{\varepsilon}_{zz}\right) $$

where *i* denotes L or Γ valley, *a*_*c*_ and *a*_*v*_ are the conduction and valence band deformation potential, respectively, and *b* is the shear deformation potential. ${\varepsilon}_{xx}={\varepsilon}_{yy}=\varepsilon =\left(\frac{a_s-{a}_{\mathrm{l}}}{a_{\mathrm{l}}}\right)$ is the in-plan strain and ${\varepsilon}_{zz}=-2\frac{C_{12}}{C_{11}}{\varepsilon}_{xx}$ is the strain in the growth direction. *a*_*s*_ and *a*_*l*_ are respectively the lattice parameter of the substrate and the strained layer. *C*_11_ and *C*_12_ are the stiffness constants.

The binary and ternary alloy material parameters are derived from those of Ge, Si, and Sn by linear interpolation. These parameters are taken from Reference [11].

The composition-dependent strained bandgaps can be evaluated by adding the corresponding strain-generated energy shifts to the unstrained material’s bandgap given in Eq. (3) for GeSn and Eq. (4) for GeSiSn:

3 $$ {E}_g^i\left({\mathrm{Ge}}_{1-{X}_d}{\mathrm{Sn}}_{X_d}\right)=\left(1-{X}_d\right){E}_g^i\left(\mathrm{Ge}\right)+{X}_d{E}_g^i\left(\mathrm{Sn}\right)-{b}^i{X}_d\left(1-{X}_d\right) $$

4 $$ {\displaystyle \begin{array}{l}{E}_g^i\left({\mathrm{Ge}}_{1-{x}_b-y}{\mathrm{Si}}_y{\mathrm{Sn}}_{x_b}\right)=\left(1-{x}_b-y\right){E}_g^i\left(\mathrm{Ge}\right)+{x}_b{E}_g^i\left(\mathrm{Sn}\right)+{yE}_g^i\left(\mathrm{Si}\right)-{b}_{\mathrm{Ge}\mathrm{Sn}}^i{x}_b\Big(1-{x}_b-\\ {}y\Big)-{b}_{\mathrm{Si}\mathrm{Sn}}^iy\left(1-{x}_b-y\right)-{b}_{\mathrm{Ge}\mathrm{Si}}^i{x}_by\end{array}} $$

where *b* is the corresponding bandgap bowing parameter of the binary alloys summarized in Table 1.

**Table 1:** Binary alloy’s bandgap bowing parameters in eV

	*b*_GeSn_	*b*_GeSi_	*b*_SiSn_
Γ (eV)	2.92 [31]	0.21 [32]	13.2 [33]
L (eV)	0.87 [31]	0.335 [32]	2.124 [32]

To determine the carriers’ confined states and deduce interband transition energies, the single-band effective mass Schrödinger equation has been solved in Cartesian coordinates by finite element method provided by COMSOL Multiphysics software [25]:

5 $$ -\frac{{\mathrm{\hslash}}^2}{2}\nabla \left(\frac{1}{m^{\ast}\left(\overrightarrow{r}\right)}\mathrm{\nabla \uppsi}\left(\overrightarrow{r}\right)\right)+V\left(\overrightarrow{r}\right)\uppsi \left(\overrightarrow{r}\right)=E\uppsi \left(\overrightarrow{r}\right) $$

*E* represents the carrier’s energy, and ψ is the corresponding wave function. m* is the carrier’s effective mass, *ћ* is the reduced Planck constant, $\overrightarrow{r}$ is the three-dimensional coordinate vector, and *V* is the carrier’s confinement potential (band discontinuity). To simplify the calculation procedure of the QD electronic structure, we have adopted the constant strain approximation [26, 27] instead of the computationally expensive atomic simulation approach that obviously could give more precision in the strain distribution profile [28, 29]. Indeed, we consider the carriers confining potential in the compressively strained QD to be sufficiently deep to minimize the impact of the strain non-uniformity on the electron confined states [27]. Furthermore, the conduction band edges, which are the most important parameters in this work, allowing to study the bandgap directness, are only shifted by the hydrostatic strain being the less sensitive to the strain non-uniformity especially when a relatively low lattice mismatch is considered [30].

## Results and Discussion

**Fig. 1:**
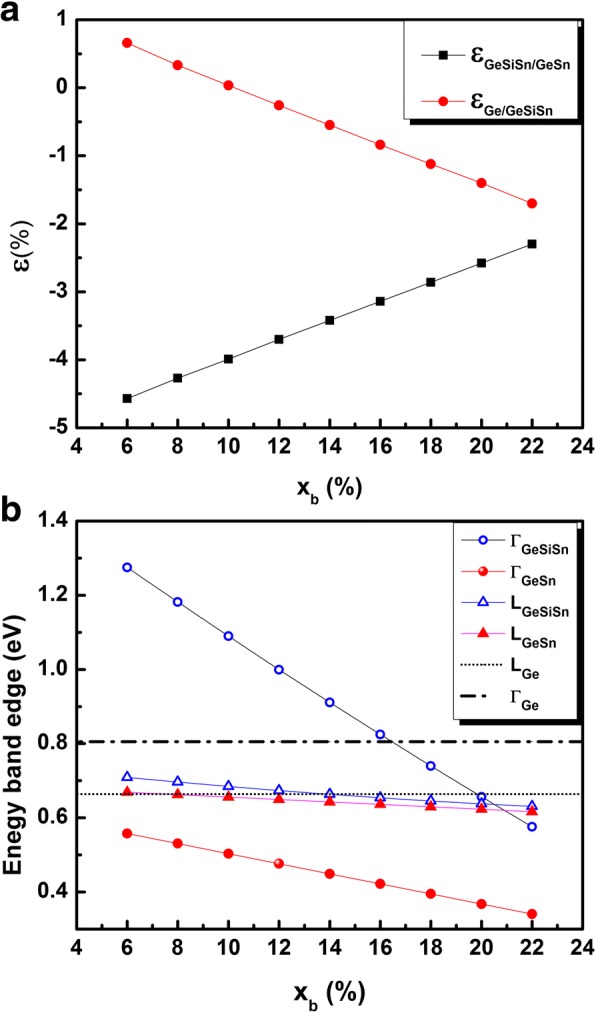
**a** Lattice mismatch between Ge_0.65-*xb*_Si_0.35_Sn_*xb*_ and Ge (filled circles) and between Ge_0.72_Sn_0.28_ and Ge_0.65-*xb*_Si_0.35_Sn_*xb*_ (filled squares) as a function of *x*_*b*._ **b** Band edges at L and G valleys for Ge_0.65-*xb*_Si_0.35_Sn_*xb*_, Ge_0.72_Sn_0.28_, and Ge as a function of *x*_*b*_

Since we are mainly concerned by the impact of the strain around the GeSn QD, the Sn composition of the QD is fixed at 28% and the Si composition of the GeSiSn at 35%; the study is therefore focused on the impact of the Sn barrier composition (*x*_*b*_) variation between 6 and 22%. The resulting in-plan strain either in the GeSiSn layer or in the GeSn QD is given in Fig. 1a.

The in-plan strain in the two-dimensional layer of GeSiSn material varies between 0.6% (*x*_*b*_ = 6%) and − 1.7% (*x*_*b*_ = 22%). We suppose that this layer remains pseudomeorphically strained allowing to keep the designed structure experimentally realizable. The GeSn is chosen to be compressively strained within the GeSiSn surrounding material with a lattice mismatch ranging from − 2.3 to − 4.5% ensuring favorable conditions to the formation of self-organized GeSn QD.

Figure 1b shows the dependence of the strained bandgap at L and Γ points from Ge_0.72_Sn_0.28_ and Ge_(0.65-*xb*)_Si_0.35_Sn_*xb*_ as a function of *x*_*b*_. The Γ valley of Ge_0.72_Sn_0.28_ material remains below the L valleys, testifying its type I for the whole investigated range of tin barrier composition. Meanwhile, when the electron confinement is taken into account, the effective bandgap increases and the QD size effect becomes decisive [18] especially for highly strained QD. Indeed, in the presence of quantum confinement, the ground state energy should be considered instead of the minimum of the Γ band. Accordingly, smaller size QD are expected to have higher confined energy levels in the Γ valley that may exceed the L valley (and/or ground state electron energy level in the L valley). So, it is important to investigate the QD size’s range obeying the specific directness criteria.

**Fig. 2:**
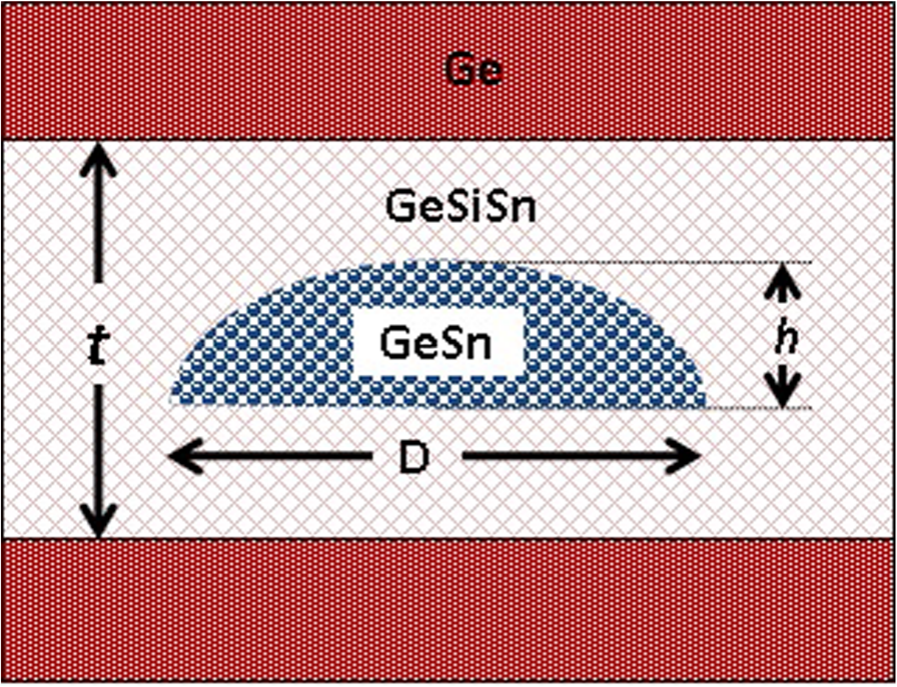
Schematic presentation of the modeled GeSn QD of height (*h*) and diameter (*D*) within GeSiSn strain-reducing layer in Ge matrix

The modeled structure is schematically presented in Fig. 2. The Ge_0.72_Sn_0.28_ QD is considered to have a dome shape with a circular base of diameter *D* ranging from 15 to 40 nm and fixed height to diameter ratio equal to 0.25. The QD is positioned inside 15-nm-thick GeSiSn layer having a Si composition of 35% and a tunable Sn composition. This structure is supposed to be formed on Ge-buffered substrate and capped with Ge layer.

**Fig. 3:**
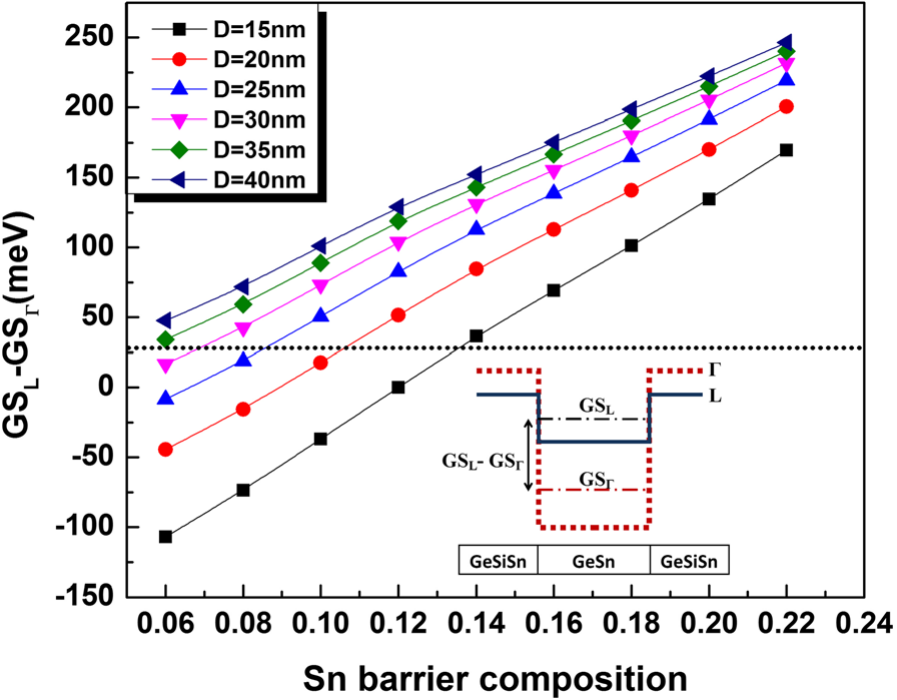
Directness parameter (GS_L_-GS_Γ_) variation as a function of the Ge_0.72_Sn_0.28_ QD size and Sn composition of the Ge_0.65-*x*_Si_0.35_Sn_*x*_ surrounding layer. The dotted line indicates the thermal energy at room temperature. The inset represents a schematic definition of the directness parameter

To ensure consistent QD design for better light-emitting device operation, a suitable directness parameter taking into the energy spacing between the lowest QD confined energy level position in L and G valleys has been introduced [18]. This parameter is denoted by GS_L_-GS_Γ_ and should be higher than the room temperature thermal energy to avoid carriers’ loss by thermal activation, where GS_L_(GS_Γ_) represents the electron ground state energy level in the L valley (Γ valley) with respect the valence band maximum. The evaluation of GS_L_-GS_Γ_ is schematically illustrated in the inset of Fig. 3.

The calculation of the GeSn QD electron energy levels in Γ and L valleys for different diameters as a function of the Sn composition in GeSiSn allows to obtain the corresponding directness parameter (GS_L_-GS_Γ_). The results are plotted in Fig. 3. For a given *x*_*b*_, the value of GS_L_-GS_Γ_ is mainly governed by the QD size. Accordingly, the smaller dots having obviously higher confined energy states require lower bandgap energy through strain reducing to fulfill the directness criteria. As shown by Fig. 3, bigger dots (*D* > 25 nm) satisfy GS_L_-GS_Γ_ > 26 meV for *x*_*b*_ higher than 8%. However, efficient direct bandgap from small-size QD is found to be ensured for higher values of *x*_*b*_ (*x*_*b*_ ≥ 14% for *D* = 15 nm).

**Fig. 4:**
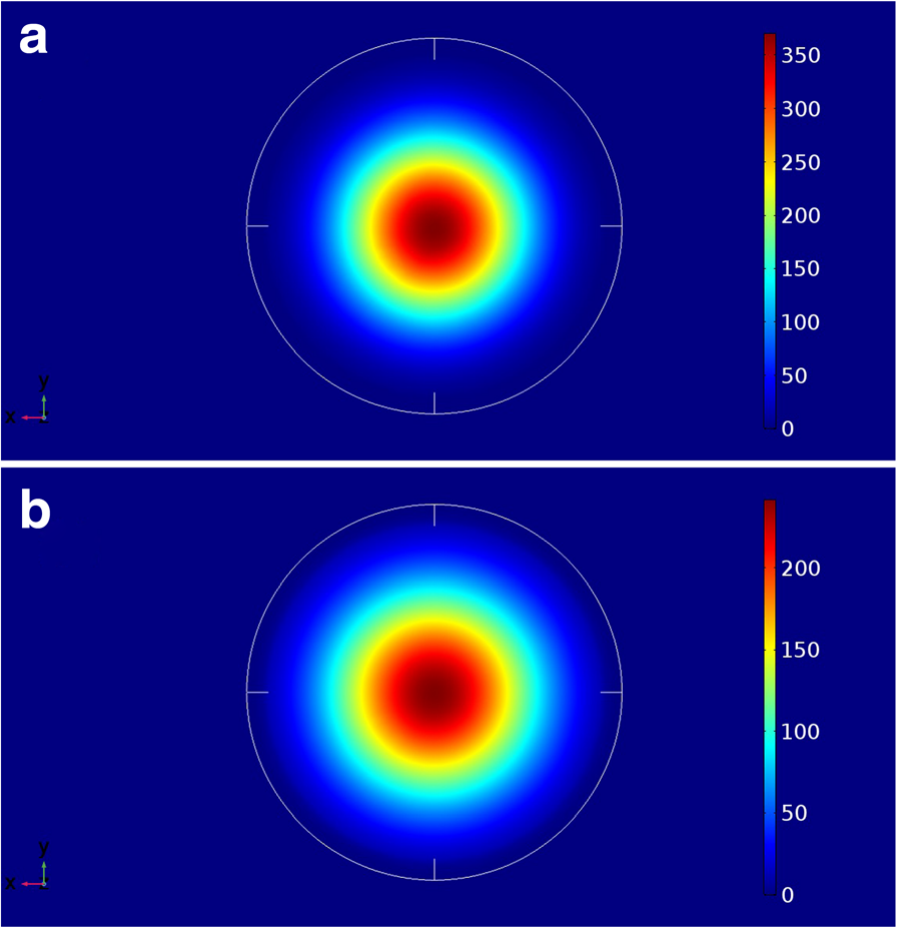
Squared electron ground state wave function for 35-nm-diameter Ge_0.72_Sn_0.28_ QD for **a** *Xb* = 6% and **b** *Xb* = 22%

Within the adopted parameters in this work, and especially the binary materials’ bowing parameters, the increase of the Sn content of the GeSiSn material reduces the stain around the QD and reduces also the surrounding material bandgap. Indeed, as shown in Fig. 1b, the increase of *x*_*b*_ from 6 to 22% reduces the conduction band discontinuity at Γ valley from 0.72 eV down to 0.23 eV. Indeed, as shown in Fig. 4, where the squared wave function ${\left|\uppsi \left(\overrightarrow{r}\right)\right|}^2$ of the ground state electron in quantum dots of diameter 35 nm is shown in the xy plan for Sn barrier composition of 6% and 22%, the electrons are found to be fully localized inside the QD regardless of the barrier composition (conduction band’s discontinuity). The strongly confined electrons indicate higher reliability of the investigated QD as an active medium for light emitters on Si substrate.

**Fig. 5:**
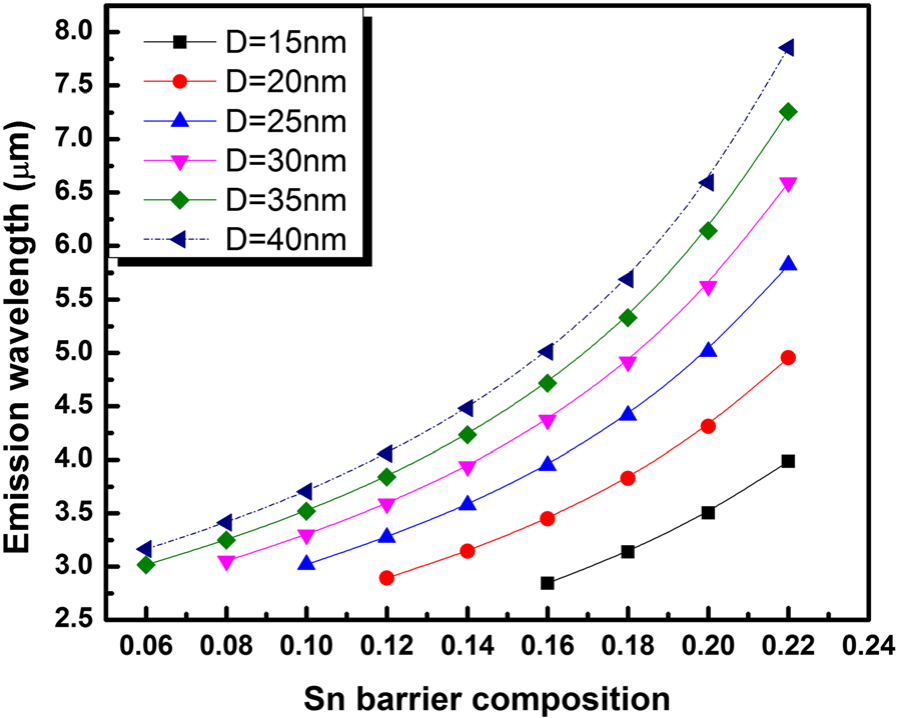
Room temperature ground state emission wavelength from direct bandgap Ge_0.72_Sn_0.28_ QD as a function of size and Sn composition of the Ge_0.65-*xb*_Si_0.35_Sn_*xb*_ surrounding layer

By limiting the QD sizes for a given *x*_*b*_ to those engendering efficient direct bandgap emission, we have appraised the QD ground state interband emission wavelength. The results are shown the Fig. 5, where the emission wavelength is plotted against *x*_*b*_ for different QD sizes. It is worth noting that the biggest QD size considered in this work (*D* = 40 nm) has shown small energy separation between the electron ground state and first excited state (below 26 meV) and has therefore been ignored from this study. Nonetheless, the evaluated emission wavelength as a function of *x*_*b*_ has been kept in Fig. 5 with a dotted line.

The wavelength range projected to be covered by the proposed QD design ranges from 3 up to 7 μm. The yielded range is extremely important for gas sensing application. The experimental implementation of this structure could offer the opportunity to cover, for the first time, the whole mid-IR range with a fully compatible material with existing microelectronic technology paving the way to new perspectives in CMOS compatible QD based mid-IR optoelectronics.

## Conclusions

GeSn QD in GeSiSn strain engineering layer on Ge matrix have been investigated as a function of QD size and the lattice mismatch with surrounding material. Reducing the strain around the GeSn QD by varying the Sn composition of GeSiSn barrier material is found to enhance the direct bandgap type I emission wavelength from 3 up to 7 μm. The designed structure opens new perspectives in mid-IR light emitter fully compatible with Si technology.

## Abbreviations

CMOS: Complementary metal-oxide-semiconductor
GS_L_: Ground state electron level in L valley
GS_Γ_: Ground state electron level in Γ valley
QD: Quantum dots
